# Potential Use of Gluconate in Cancer Therapy

**DOI:** 10.3389/fonc.2019.00522

**Published:** 2019-06-19

**Authors:** Maria E. Mycielska, Markus T. J. Mohr, Katharina Schmidt, Konstantin Drexler, Petra Rümmele, Sebastian Haferkamp, Hans J. Schlitt, Andreas Gaumann, Jerzy Adamski, Edward K. Geissler

**Affiliations:** ^1^Section of Experimental Surgery, Department of Surgery, University Hospital Regensburg, Regensburg, Germany; ^2^Metempyrosis–Data Analysis in Medicine and Information Technology, Regensburg, Germany; ^3^Department of Dermatology, University Hospital Regensburg, Regensburg, Germany; ^4^Institute of Pathology, University Hospital Erlangen, Friedrich-Alexander University Erlangen-Nuremberg, Erlangen, Germany; ^5^Institute of Pathology Kaufbeuren-Ravensburg, Kaufbeuren, Germany; ^6^Molecular Endocrinology and Metabolism, Helmholtz Zentrum München, German Research Center for Environmental Health, Neuherberg, Germany; ^7^Lehrstuhl Für Experimentelle Genetik, Technische Universität München, Munich, Germany; ^8^Department of Biochemistry, Yong Loo Lin School of Medicine, National University of Singapore, Singapore, Singapore

**Keywords:** gluconate, cancer metabolism, citrate, metabolic targeting, transporter

## Abstract

We have recently discovered that cancer cells take up extracellular citrate through plasma membrane citrate transporter (pmCiC) and advantageously use citrate for their metabolism. Citrate uptake can be blocked with gluconate and this results in decreased tumor growth and altered metabolic characteristics of tumor tissue. Interestingly, gluconate, considered to be physiologically neutral, is incidentally used in medicine as a cation carrier, but not as a therapeutically active substance. In this review we discuss the results of our recent research with available literature and suggest that gluconate may be useful in the treatment of cancer.

## Introduction

Despite intense basic and clinical cancer research, progress in controlling and curing malignancies remains slow. Surgery, chemotherapy and radiotherapy are often palliative especially for advanced cancer, and can have severe side effects. Emerging immunotherapies are showing promise, but much work still needs to be done to prove their efficacy against a wide variety of tumors, and tumors with little immunogenicity will likely not respond well to these therapies.

Intense research efforts are directed toward identifying novel anti-cancer drugs and to discover known substances with potential antineoplastic effects that have been used for indications other than cancer (1). This latter approach has the advantage that new discoveries with approved substances can be moved more quickly to clinical application. In the present review we chose to concentrate on gluconate as an interesting candidate molecule that is often used as a “carrier” for putative antineoplastic substances, therefore disguising its own potential anti-cancer activities. We raise the possibility that activities thought to be attributed entirely to the putative active antineoplastic substance, may at least in part be due to the activity of gluconate. Based on our own studies and the available literature we propose that gluconate should be studied as an anti-cancer drug alone or in combination with chemotherapy. This idea is particularly attractive since gluconate use as an excipient is approved by the FDA and is considered to have little in the way of any side effects. Herein, we also present an interpretation of the existing data explaining why gluconate could also be useful in the diagnosis of cancer.

## Gluconate

Gluconate is a glucose derivative, existing as a salt of gluconic acid known to chelate divalent metals; gluconic acid is found naturally in fruits, honey and wine. As a membrane impermeant anion, gluconate is often used in biomedical research as Cl^−^ substitute (2, 3). In medicine, gluconate is used most commonly as a biologically neutral carrier of Zn^2+^, Ca^2+^, Cu^2+^, Fe^2+^, and K^+^ to treat relevant ion deficiencies. Furthermore, gluconate is also combined with other drugs such as chlorohexindine, which is used as an antiseptic in surgery (4) or dental care (5); sodium antimony (stibogluconate) is used to treat leishmaniasis (6). Importantly, however, while gluconate is exploited for these purposes in medicine, it is not considered to be physiologically active and has not been studied for its own therapeutic effects.

## Mechanism of Gluconate Suppression of Cancer Growth

**Figure 1:**
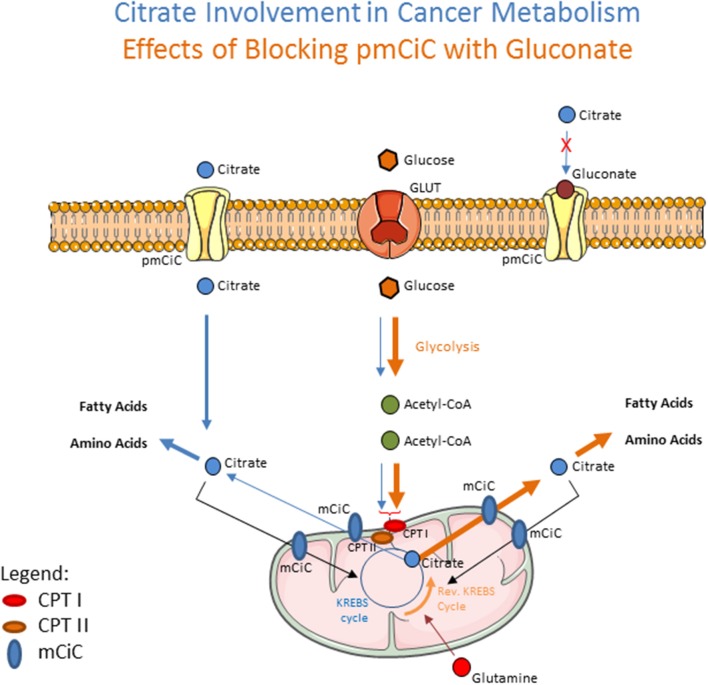
Schematic drawing showing the effects of extracellular citrate on cancer cell metabolism (blue arrows). Extracellular citrate is taken up by the cells through the plasma membrane citrate transporter (pmCiC). Its uptake reduces the need of mitochondrial-derived citrate (transported from mitochondria through mCiC) and is mainly used in cytoplasm to support fatty acid and amino acid synthesis. When the pmCiC is blocked with gluconate (brown arrows), extracellular citrate cannot get through the plasma membrane of cancer cells resulting in increased use of glycolysis to support mitochondrial citrate synthesis. CPTI and II (Carnitine palmitoyltransferase I and II) depict AcetylCoA transport mechanism. The thickness of lines reflects the intensity of pathway use.

How can the hypothesis of gluconate's anticancer effects be rationalized scientifically? Although citrate is considered a central substrate in cancer cell metabolism, its source remains debatable. Interestingly, several recent metabolomic studies indicate a correlation between metastatic disease and decreased level of citrate in blood, tissues and urine of cancer patients (7–11), to which an increased need of the cancer for citrate could contribute. It is also possible that under these pathophysiological conditions decreased citrate could be accounted for by increased liver metabolism, especially regarding fatty acid synthesis (12). Since hepatocytes express Na^+^-dependent citrate transporter (NaCT), increased citrate uptake from blood could additionally be associated with increased metabolic liver function (13). We have recently put forward a hypothesis in which cancer cells take up extracellular citrate and use it to support their metabolism, especially fatty acid synthesis (14). Our work shows that cancer cells take up extracellular citrate through the plasma membrane citrate transporter (pmCiC) belonging to the SLC25 family and that expression of this transporter is mainly restricted to cancer cells (14). We have also shown that cancer cell metabolism benefits from extracellular citrate uptake by decreasing its mitochondrial activity and in consequence ROS synthesis, thereby reducing cancer cell requirements for extracellular glucose supply. Importantly, we have discovered that gluconate is a competitive and irreversible blocker of the pmCiC, thus decreasing the uptake of extracellular citrate by cancer cells, and inhibiting human tumor growth in immunodeficient mice (summarized in Figure 1). This observation together with largely cancer cell-specific expression of pmCiC provides a mechanistic explanation for why gluconate could be partially responsible for the increased anti-cancer effects observed when used with other therapeutics, like Zn^2+^ and Ca^+2^. For instance, therapeutic remissions observed when using Zn^2+^ gluconate for ocular melanoma and acute lymphocytic leukemia could actually be related to the presence of gluconate (15, 16), as will be detailed later in this review. Of course some of the observed effects are likely due to ions carried by gluconate, such as increased immune activity associated with Zn^2+^, or protection against neuropathy known to be due to Ca^2+^. Furthermore, from a diagnostic standpoint, our research finding that gluconate binds to the pmCiC provides an explanation for why 99mTc gluconate (technetium-99m, used in medicine as a radioactive tracer due to its short half-life), is guided specifically to malignant cancer entities. The following part of this article presents available data related to the use of gluconate in cancer treatment and diagnosis. We include an interpretation of the potential gluconate effects based on our own studies.

## Stibogluconate in Cancer Immunotherapy

Stibogluconate (sodium antimony gluconate, sodium stibugluconate, pentostam, stibium Sb gluconate) is an anti-parasitic drug that incorporates gluconate into the drug substance. It belongs to the anti-leishmaniasis group of drugs called pentavalent-(V)-antimony compounds in which antimony (Sb) is used (17–19). It has been suggested that stibogluconate acts through immune system stimulation, mainly by increasing the amount of peripheral IFN-γ^+^ T cells (20). Importantly, this increased immune response has been observed in mice (21), as well as in humans (22). Because of its stimulating effect on the immune system, the drug was considered a good candidate for cancer research. Indeed, application of stibogluconate *in vivo* in mice with implanted human tumor cells consistently resulted in either significant tumor growth inhibition or total tumor regression. Human renal cell carcinoma growth was significantly inhibited in mice using stibogluconate (21). Similar results were obtained when mice bearing human melanoma cells were treated with this substance (23). Interestingly, other pentavalent antimony compounds, not incorporating gluconate, including triphenylantimony-(V)-polyamines and monomeric species, showed limited effects on cancer cells [reviewed by (24)]. Furthermore, trivalent-(III)-antimony, not associated with gluconate, has not shown promising anti-tumor effects (applied in the form of organometallic compounds such as diphenylantimony(III) thiolates). Application of these compounds against leukemia in mice did not give a substantial survival advantage and showed high dosage-dependent toxicity (25).

Why therefore are some antimony forms effective against cancer (stibogluconate), and others are not? Possible explanations include differences in transport mechanism of alternative forms of antimony, which are still not well-defined. While transport of antimony-(III) from the extra- to intracellular space is thought to occur through aquaglyceroporins and hexose transporters, proteins involved in the uptake of antimony-(V) have not been identified [reviewed by Maciaszczyk-Dziubinska et al. (26)]. What is known is that antimony-(V) is taken up by cells at a lower rate than antimony-(III) and is metabolically reduced within the cell to antimony-(III) through the action of glutathione; this process occurs before cytotoxic effects are apparent (27, 28). Interestingly, experiments *in vitro* (29) show that cancer cells that are resistant to antimony-(III) action, are sensitive to stibogluconate [antimony-(V) treatment]. Together, these reports indicating that cells resistant to antimony-(III) are not resistant to stibogluconate [antimony-(V)] lead us to the hypothesis that gluconate could be an, if not the, active anti-cancer substance in this scenario.

## Other Studies and Trials Involving Gluconate

### PD-1

PD-1 (programmed death 1) receptors on T lymphocytes provide an immune checkpoint that functions to prevent over-activation of the immune system to antigenic challenges. Importantly, malignant tumors often evolve to express programmed death-ligand 1 (PD-L1), which is the ligand for PD-1 that suppresses T cell activation (30) and thereby prevents the immune system from destroying cancer cells. Targeting PD-1 has indeed proved to be an effective target in cancer therapy to boost antitumor immunity (30–33). Clinical trials using anti-PD-1 antibody have produced positive results in a subgroup of patients with certain types of cancer (34–36). Unfortunately, this treatment is often accompanied by severe side-effects, including autoimmune reactions (37, 38). In an attempt to decrease these adverse effects, a self-degradable microneedle patch was developed to deliver the anti-PD1 antibody directly to melanoma lesions. To facilitate gradual release of the anti-PD-1-antibody and to control release into the cancer site, a system was invented to encapsulate antibody in pH-sensitive dextran nanoparticles. To decrease pH locally, glucose oxidase converting blood glucose to gluconic acid was encapsulated together with the anti-PD-1 antibody in dextran sensitive nanoparticles. Locally increased levels of gluconic acid in blood (decreasing pH) were expected to induce destruction of dextran nanoparticles allowing for the release of the antibody. Interestingly, addition of this enzyme significantly increased survival of mice with B16F10 melanoma cells and decreased tumor growth as compared to patches with antibody, but no glucose oxidase (39). We raise the possibility that the additional anticancer effect was at least in part due to the locally increased gluconate level, thus blocking extracellular citrate uptake by cancer cells.

### Zn^2+^ Gluconate

Encouraging results with Zn^2+^ gluconate used as an adjuvant therapy to standard chemotherapy have been obtained when treating children with acute lymphocytic leukemia, with Zn^2+^ being applied to stimulate the immune system. Administration of Zn^2+^ gluconate significantly improved the effects of chemotherapy. While 30 days of chemotherapy application usually leads to bone marrow blast reduction to 3–5% when applied together with Zn^2+^ gluconate, it resulted in undetectable numbers of blasts. In this case Zn^2+^ gluconate was administered 2x per day from the start of chemotherapy until the end of 3 years of maintenance therapy (16). This treatment resulted in a complete bone marrow remission and normal hematopoietic function in all 13 children. Interestingly, application of 6-mercaptopurin accompanied by Zn^2+^ sulfate used to treat acute lymphoblastic leukemia did not produce this effect, but notably the application was over a significantly shorter time period (40). Nonetheless, this result is in keeping with our hypothesis regarding the potential anti-cancer effect of gluconate.

Very promising results were also obtained from the phase 1 trial of an IRX-2 (mmunostimulatory drug comprising a variety of cytokines derived from lymphocytes) regimen in patients with squamous cell carcinoma of the head and neck (41). Out of 8 patients only 2 showed progressive tumors after 21 days of treatment. Interestingly, IRX-2 treatment in this trial was accompanied by a daily intake of Zn^2+^ gluconate. Zn^2+^ was added because of its known effects on the immune system, reflected by Zn^2+^ deficiencies associated with decreased numbers and activity of different types of immune cells (42). Intriguingly, zinc deficiency has been correlated with several types of cancer (43). The fact that gluconate was used as part of the Zn^2+^ formulation in the IRX-2 study presents the possibility that pmCiC blockade could also have contributed to the anti-cancer effects that were observed.

It is also important to discuss one particular case report describing successful long-term treatment of a liver metastasis from ocular melanoma (15). In this case, intake of disulfiram was accompanied by oral administration of Zn^2+^ gluconate 3x per day and the treatment regimen of reduced-dose disulfiram with full dosing of Zn^2+^ gluconate was continued for several years (similar to the case of acute childhood leukemia described above). In our view, there were 3 substances in this case used to treat the ocular melanoma that could account for the success of the treatment: disulfiram, Zn^2+^ and gluconate, or a combination of these. The main substance considered to have anti-cancer activity in this study was disulfiram, while Zn^2+^ was intended to enhance its anti-cancer activity. However, none of the published results from clinical studies using disulfiram without heavy metal combined with gluconate as the adjuvant therapy confirmed its anti-cancer activity. In a recent study involving patients with non-small cell lung carcinoma receiving cisplatin together with disulfiram as adjuvant therapy (not accompanied by heavy metal administration), no difference in the tumor response rate was observed with chemotherapy plus or minus disulfiram (44). Similarly, a study on cisplatin-sensitive malignancies reported no significant difference in the time to progression or median survival time between patients treated with cisplatin and disulfiram, or cisplatin only (45). No improvement of non-metastatic recurrent prostate cancer was observed when different doses of disulfiram alone were applied (46). Therefore, we raise the possibility that in the case of tumor response with ocular melanoma liver metastasis, gluconate should be considered as a potential mediator of anti-cancer activity.

### Ca^2+^ Gluconate

Gluconate has also been applied through injections to carry Ca^2+^ to prevent neuropathy in patients receiving oxaloplatin treatment. Oxaliplatin increases extracellular levels of oxalate which chelates Ca^2+^. Decreased Ca^2+^ levels have been postulated to induce prolonged activation of K^+^-independent voltage-gated Na^+^ channels resulting in stress and mitochondrial damage of nerve cells leading to neuropathy (47). To increase extracellular Ca^2+^ levels and to prevent unwanted side effects during oxaliplatin treatments for colorectal cancer, 1 g of Ca^2+^ gluconate was injected before and after oxaliplatin infusion. Although there is a general consensus that Ca^2+^ treatment does not affect the efficiency of oxaliplatin treatment, there are conflicting results regarding its use in decreasing neuropathy from no improvement (48), decrease in acute sensory neuropathy (47), to a significant reduction in neuropathy (49). While the first two studies were performed on a small number of patients (~20), the third study included a larger cohort of 161 patients. Importantly, not only a significantly better tolerance to oxaliplatin treatment was observed in the patient group injected with Ca^2+^ gluconate, but the anti-tumor response rate was increased from 35% (control) to 45% in the Ca^2+^ gluconate pretreated group (49). Since the studies were performed to test the effect of Ca^2+^ infusions on neuropathy, the conclusions regarding any potential anti-tumor effects of Ca^2+^ or gluconate can only be considered speculative at this point.

## Gluconate in the Diagnosis of Cancer

Increased uptake of radioactive glucose has been used in positron emission tomography (PET) analysis as an indicator of cancerous lesions. A drawback of this technique is that glucose uptake is not only associated with cancer, it is also typical of inflamed tissues. To overcome this problem several different labeled substances have been studied as potential diagnostic markers. Additionally, a search has been underway to find markers that distinguish between metastatic and non-metastatic lesions (50–53). These studies became particularly interesting with the development of radiolabeling techniques that led to the synthesis of new labels. One of them, presently used in many medical diagnostic tests due to its relatively high radioactivity and a short half-life, is technetium 99 (99mTc). While initial studies indicated 99mTc-glucoheptonate as a promising substrate that could help distinguish between benign and metastatic lesions in the lung, more detailed studies showed that the specificity was low. Therefore, other glucose derivatives such as gluconate have been considered (54). The use of 99mTc-labeled gluconate was efficient in the diagnosis of tumors of different origin, including the detection of intracranial metastases (55). Another trial revealed that labeled gluconate can be successfully used to distinguish between benign and malignant lesions in the lung, showing significantly better sensitivity and accuracy than radiography (54). Similarly, promising results were obtained when 99mTc-diethylenetriaminepentaacetic acid radionuclide-based angiography was combined with 99mTc-gluconate renal venography. This technique was used for the specific detection of malignant lesions in kidney and bladder cancer (56), and showed that the applied radiolabeled substances led to proper localization of the lesions and accurate assessment of malignancy. Additionally, it was observed that the use of 99mTc gluconate significantly reduced background noise as compared to other substances, and this was considered to be the major advantage (55). Our recent publication (14) finally sheds light on why gluconate binding to tumor is specific. We show that gluconate binds irreversibly to the pmCiC expressed on cancer cells (14), and the expression of pmCiC is high in metastatic lesions and more advanced disease. Therefore, labeled gluconate has the potential to distinguish between different tumor grades, although this will require additional detailed studies.

Importantly, it has been observed that the concentration of 99mTc-gluconate reaches its highest level in the kidney 1–2 h after injection (57). Moreover, there was no signal observed from adjacent organs/tissues, indicating a lack of 99mTc-gluconate uptake by healthy cells. The lack of 99mTc-gluconate uptake by healthy organs and fast clearance through the kidney was later confirmed (58). These features suggest that the potential diagnostic use of gluconate would likely have few if any side effects.

To summarize, early studies indicate that labeled gluconate is useful in cancer diagnostics because of its ability to distinguish between benign and metastatic lesions. Interestingly, until our recent research findings (14), there was no explanation as to why malignant cancers have an affinity for gluconate.

## Treatment Options

Based on our own research and the available literature data we suggest that gluconate should be studied in the context of anti-cancer treatment. Electrophysiological experiments revealed that gluconate blocking of citrate transport in cancer cells is irreversible and the effect increases with every subsequent application (14). Moreover, half-life of plasma membrane proteins is relatively long and in the case of pmCiC it has been established to be around 48h (14). Based on these data one could speculate that smaller doses of gluconate taken frequently should be more effective than large bolus doses of gluconate given e.g., IV. However, since nothing is known about oral gluconate pharmacokinetics, dosing studies should be performed before considering cancer treatment options. The possible effectiveness of oral gluconate application would be consistent with the successful treatment observed with the previously discussed ocular melanoma (15) and acute lymphocytic leukemia (16) cases where gluconate was given orally in small doses for several years.

Blocking of the pmCiC could also have indirect positive effects in cancer treatment. Indirect activity is possible since blocking citrate uptake in cancer cells *in vivo* has been shown to increase ROS synthesis (14). Indeed, increasing ROS synthesis to promote cancer cell apoptosis is already part of the action of several chemotherapeutics e.g., doxorubicin, daunorubicin or epirubicin (59, 60). Therefore, pretreatment of cancer patients receiving chemotherapy with gluconate should be explored for beneficial effects by decreasing cancer cell resistance and increasing efficiency of chemotherapy. For this purpose, single large doses of gluconate given 24 and 48 h before administering chemotherapy (49) should be considered.

With regard to potential side effects, there are currently no reports of gluconate specifically exerting any adverse events in patients. Side effects implicated when taking e.g., Zn^2+^ gluconate have been associated with increased Zn^2+^ absorption, rather than gluconate action. Nonetheless, a safety assessment will need to be made if gluconate does enter testing as an anti-cancer agent.

## Conclusions

In this review we have presented several independent clinical and basic research studies in which gluconate was used incidentally together with other substances to treat cancer. Indeed, gluconate is considered to be physiologically neutral and therefore was not administered in these cases as the drug substance. Since putative anticancer agents like those described in this review tend to show positive effects mainly when combined with gluconate, an argument can be made for gluconate actually playing a role in reported anticancer activities. These examples, together with our latest research results showing that gluconate blocks citrate transport into cancer cells and inhibits tumor growth in mice, raises the intriguing hypothesis that gluconate use may offer an interesting new diagnostic and treatment option in cancer research.

## Author Contributions

All authors listed have made a substantial, direct and intellectual contribution to the work, and approved it for publication.

### Conflict of Interest Statement

MEM, PR, and EG are co-inventors on a patent application (EP15767532.3 and US15/514,255) related to plasma membrane citrate transporter in the diagnosis and treatment of cancer, filed by the Universitätsklinikum Regensburg. The remaining authors declare that the research was conducted in the absence of any commercial or financial relationships that could be construed as a potential conflict of interest.
